# Quantitative RT-PCR Detection of Hepatitis A Virus, Rotaviruses and Enteroviruses in the Buffalo River and Source Water Dams in the Eastern Cape Province of South Africa

**DOI:** 10.3390/ijerph9114017

**Published:** 2012-11-05

**Authors:** Vincent Nnamdigadi Chigor, Anthony Ifeanyi Okoh

**Affiliations:** Applied and Environmental Microbiology Research Group (AEMREG), Department of Biochemistry and Microbiology, University of Fort Hare, Private Bag X1314, Alice 5700, Eastern Cape, South Africa; Email: vnchigor@yahoo.com

**Keywords:** real-time RT-PCR, enteric RNA viruses, surface waters, detection, quantification, hepatitis A virus, rotaviruses, enteroviruses

## Abstract

Human enteric viruses (HEntVs) are a major cause of water-related diseases. The prevalence of hepatitis A virus (HAV), rotaviruses (RoV) and enteroviruses (EnV) in Buffalo River waters was assessed quantitatively over a period of 12 months (August 2010 to July 2011). Seventy-two samples were collected from six sites, including three dams, and concentrated using the adsorption-elution method. Viral RNA was extracted using a commercial kit, and the viruses were quantified by real-time quantitative reverse transcriptase PCR (RT-qPCR). Two or more viruses were detected in 12.5% of the samples. HAV was detected in 43.1% of the samples and in significantly (*p* < 0.05) varying concentrations of 1.5 × 10^1^–1.9 × 10^5^ genome copies/L compared to RoV and EnV, while RoVs were detected in 13.9% of samples, with concentrations ranging from 2.5 × 10^1^–2.1 × 10^3^ genome copies/L, and EnV were detected in 9.7% of the samples, with concentrations ranging from 1.3 × 10^1^–8.6 × 10^1^ genome copies/L. Only HAV was detected at all the sites, with the Bridle Drift Dam recording significantly higher (*p* < 0.05) concentrations. The presence of enteric viruses in Buffalo River may constitute public health risks and the incidence of HAV at all the sites could reflect both the epidemiological status of hepatitis A and HAV persistence in the water environments.

## 1. Introduction

Microbial contamination of water remains a problem of global concern [1,2] and lack of safe water and poor sanitation are important risk factors for mortality and morbidity, including diarrhoeal diseases, especially in the developing world [1,3,4]. A recent report by the WHO/UNICEF Joint Monitoring Programme for Water Supply and Sanitation shows that 884 million people, almost all of them in the developing regions of the World, still do not get their drinking-water from improved sources. Sub-Saharan Africa accounts for over a third of that number. The report further reveals that seven out of ten people without improved sanitation live in rural areas, and that worldwide, 37% of people not using improved source of drinking water live in Sub-Saharan Africa [5]. South Africa is a semi-arid, water-stressed country sourcing water largely from rivers and dams for the production of drinking water, as well as for agricultural and recreational purposes [6]. However, such surface waters are vulnerable to faecal contamination [7,8,9].

Human enteric viruses (HEntVs) are excreted in high concentrations (10^5 ^to 10^13^/g faeces) in the faeces of infected persons and have great potential to pollute water sources [10,11]. It has been estimated that 30–90% of waterborne disease outbreaks worldwide are caused by HEntVs [12]. HEntVs are transmitted mainly by the faecal-oral route, either direct from person-to-person or via consumption of contaminated food or water [13]. Over 140 enteric viruses are known to infect humans and they are considered to be emerging waterborne pathogens because of their high stability in the environment and resistance to current water treatment processes [12,14]. HEntVs belong to the families *Picornaviridae* (polioviruses, enteroviruses, coxsackieviruses, echoviruses and hepatitis A virus), *Caliciviridae* (noroviruses and sapoviruses), *Astroviridae* (astroviruses), *Reoviridae* (rotaviruses), *Hepeviridae* (hepatitis E virus) and *Adenoviridae* (adenoviruses) [15].

Aside from the adenoviruses that have a DNA genome, most health-significant waterborne viruses have RNA genomes. These enteric RNA viruses have low infective doses [16] and among the diseases caused by them are epidemic gastroenteritis, hepatitis, paralysis, meningitis and myocarditis [11,17,18]. Worldwide, while rotavirus and hepatitis A virus are the leading causes of epidemic gastroenteritis and acute hepatitis, respectively, enteroviruses are major causes of paralysis, meningitis and myocarditis [11,19,20,21].

Hepatitis A virus (HAV), a 27- to 32-nm non-enveloped, small, single-stranded RNA virus and the only member of the *Hepatovirus* genus [19] is the aetiological agent of hepatitis A which is hyperendemic in South Africa [22,23]. Rotavirus (RoV) is a non-enveloped, double-stranded RNA virus and the leading cause of severe diarrhoea among infants and young children, with an estimated 611,000 deaths from rotavirus infection per year worldwide [24], and almost half of all deaths worldwide are estimated to occur in Africa [25,26]. Enteroviruses (EnV) are non-enveloped, single-stranded RNA viruses that include poliovirus, coxsackieviruses, echoviruses and the numbered enteroviruses [20].

Waterborne outbreak of infections caused by these enteric RNA viruses have been reported worldwide [12,27,28,29,30], South Africa inclusive [31,32,33]. These viruses have also been detected in water sources globally [34,35,36,37,38,39,40,41]. A number of South Africa studies have also reported the detection or isolation enteric RNA viruses from water sources, including polio virus and non-polio enteroviruses [42,43,44], astroviruses [22,45], rotaviruses [46,47], and hepatitis A virus [22,23]. However, the studies that have assessed for viral agents in South Africa’s waters have occurred only in a limited number of locations and provinces, and no records exist of similar investigations in Eastern Cape Province.

Recent studies have highlighted both the drawbacks of using only bacteriological indicators and the necessity of surveillance of source waters for viral pathogens towards protection of public health [48,49,50]. Standard methods for the detection of infectious viruses in water require the use of susceptible cell lines within which the viruses can propagate and produce cytopathic effects [35,51]. However, cell culture can be time-consuming, labour-intensive and unsuitable for the detection of some nonculturable/noncytopathic enteric viruses, for which either the appropriate cell cultures are not available (e.g., Norovirus) or the growth of the viruses is limited (e.g., HAV) [52,53]. Additionally, typically there is a low density of virus in water samples [19]. However, progress has been made developing molecular techniques and polymerase chain reaction (PCR) is currently the most widely used technique for the detection of viruses in various kinds water samples due to its high sensitivity and specificity [11,12,35,54,55].

Reverse transcriptase PCR (RT-PCR) involves a step in which the viral RNA genome is reverse transcribed to a complementary DNA strand (cDNA) prior to the PCR and has been successfully used to monitor water for enteric RNA viral contamination [17,19,22,52]. Over conventional (qualitative) RT-PCR, real-time RT-PCR has the advantage of enabling sensitive and rapid the determination of concentrations of viral pathogens in environmental samples [56,57,58,59,60]. A major limitation of the PCR assays is their inability to determine the viability and infectivity of viruses detected, as the presence of viral nucleic acid does not necessarily indicate the presence of infectious viruses [61,62]. However, a recent study has demonstrated a statistical correlation between genome copy numbers and infectious enteric viral particles in wastewater samples and proposed that a cut off value of 200 genome copies could be used to indicate viral survival in environmental monitoring [63]. The use of propidium monoazide in RT-PCR (PMA-RT-PCR) has also been shown to be effective for distinguishing between infectious and noninfectious enteric RNA viruses in water samples [64]. We report the findings of our studies on the quantitative RT-PCR detection of hepatitis A virus, rotaviruses and enteroviruses in the Buffalo River, a major water resource in the Eastern Cape.

## 2. Experimental Section

### 2.1. Study Area and Samples Collection

**Figure 1:**
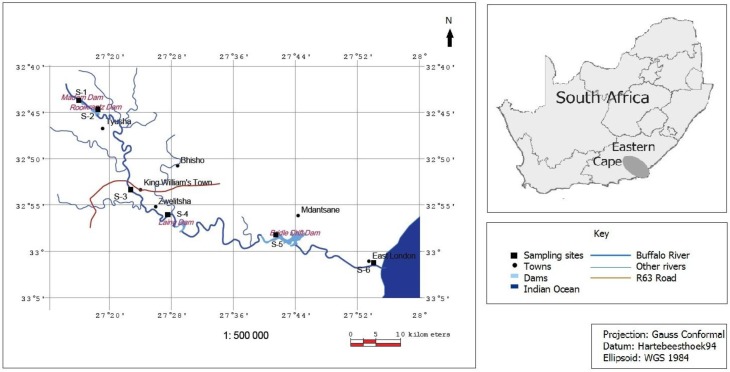
The study area and samplingsites.

Rising at an altitude of 1,200 m in the Amathola Mountains of the Eastern Cape, the Buffalo River (Figure 1) flows south-eastwards for about 126 kilometres before emptying into the Indian Ocean at East London harbour. The catchment supports about 570,000 people within its 1,287 km^2^ area [65]. Along the Buffalo River there are four dams supplying water to the urban areas of King William’s Town, Zwelitsha, Mdantsane and East London. Buffalo River is not only important as the major source for raw water abstraction; it is used also for irrigation and recreational purposes.

Six different sampling sites were identified on the river course using a GPS instrument (Garmin; eTrex Legend H). These include S-1, Maden Dam (32°44'22"S; 27°17'54"E), S-2, Rooikrantz Dam (32°45'19"S; 27°19'35"E), S-3, King William’s Town (32°53'23"S; 27°23'17"E), S-4, Eluxolzweni (32°56'16"S; 27°27'56"E), S-5, Bridle Drift Dam (32°58'30"S; 27°42'22"E) and S-6, Parkside (33°01'23"S; 27°51'31"E), in East London. From August 2010 to July 2011, water samples were collected monthly from the sites using sterile 1.75-litre screw-capped bottles and transported on ice to the Applied and Environmental Microbiology Research Group (AEMREG) laboratory at the University of Fort Hare, Alice, South Africa where they were kept at 4 °C until processing as recommended by American Public Health Association [66].

### 2.2. Concentration of Viruses in Water

The water samples were first passed through glass fibre prefilters (Millipore, County Cork, Ireland) to remove debris and minimize membrane clogging. Concentration was carried out following the adsorption-elution method of Haramoto *et al.* [67]. This method based on electrostatic interactions was reported to have shown a recovery efficiency of 56% ± 32% (*n* = 37) for surface water samples inoculated with polioviruses. Briefly, 5 mL of 250 mM AlCl_3_ was passed through an HA filter (0.45 µm pore size and 47 mm diameter; Millipore) to form a cation (Al^3+^)-coated filter, and then one litre of the river water sample was filtered through. The filter was then rinsed with 200 mL of 0.5 mM H_2_SO_4_ (pH 3.0), followed by elution of viruses with 10 mL of 1.0 mM NaOH (pH 10.8). This acid rinse removes the Al^3+^ and allows the viruses to attach directly to the negatively charged membrane. The eluate was recovered in a tube containing 50 µL of 100 mM H_2_SO_4_ (pH 1.0) and 100 µL of 100 × Tris-EDTA (TE) buffer (pH 8.0) for neutralization, followed by centrifugation using a Centriprep^®^ centrifugal filter, Ultracel^®^ YM-50 (Millipore), according to the manufacturer’s protocol. Ultracentrifugation, using a centrifuge (Model TJ-6; Beckman, Brea, CA, USA), was carried out at 2,500 rpm for 10 min. This was followed by removal of the sample that passed through the ultrafiltration membrane (about 8 mL) and further centrifugation at 2,500 rpm for 5 min to obtain a final volume of about 700 µL. Each final concentrated sample was aliquoted in 200 µL regimens and stored at −80 °C until further analysis [67].

### 2.3. Quantification of Viruses

The concentration of the viruses in the samples was determined by real-time RT-PCR using a *StepOnePlus* PCR System (OPTIPLEX 755, Applied Biosystems, Foster City, CA, USA) and following a two-step protocol involving a reverse-transcription step and complementary DNA (cDNA)-based qPCR step. The real-time procedures for HAV were based on the amplification of a fragment of the highly conserved 5′ noncoding region (5′ NCR) [56]. The rotavirus real-time RT-PCR assay utilized primers and probe that were designed to target the non-structural protein region 3 (NSP3) of rotavirus [59]. The pan-enteroviral primers and probe are specific for a 143-nucleotide portion of the 5′ untranslated region (5′ UTR) of poliovirus. This 5′ UTR region is highly conserved in non-polio enteroviruses and poliovirus types [57]. All the primers and probes used in this study (Table 1) were designed by previous investigators and synthesized by Applied Biosystems.

**Table 1:** Primers, probes, and control viruses used for the real-time RT-PCR.

Enteric virus	Primers and labelled TaqMan probe	Reference	Control virus
Hepatitis A virus	HAV68 (F): 5′-TCA CCG CCG TTT GCC TAG-3′	[56]	ATCC VR-1357; Strain PA21
	HAV240 (R): 5′-GGA GAG CCC TGG AAG AAA G-3′		
	HAV150 (P): 5′-FAM-CCT GAA CCT GCA GGA ATT AA-MGBNFQ-3′		
Rotaviruses	JVK (F): 5′-CAGTGGTTGATGCTCAAGATGGA-3′	[59]	ATCC VR-2274; Strain 248
	JVK (R): 5′-TCATTGTAATCATATTGAATACCCA-3′		
	JVK (P): 5′-FAM-ACAACTGCAGCTTCAAAAGAAGWGT-MGBNFQ-3′		
Enteroviruses	EV1 (F): 5′-CCCTGAATGCGGCTAAT-3′	[57]	Coxsackie virus A2 (ATCC VR-1550; Strain FLEETWOOD)
	EV1 (R): 5′-TGTCACCATA AGCAGCCA-3′		
	EV-BHQ (P): 5′-FAM-ACGGACACCCAAAGTAGTCGGTTC-MGBNFQ-3′		

Abbreviations: F, forward/sense; R, reverse/antisense; P, probe; FAM, 6-carboxyfluorescein (reporter dye); MGBNFQ, minor groove binder/nonfluorescent quencher.

#### 2.3.1. RNA Extraction

Viral RNA was extracted from 200 µL of the sample (eluate) using a commercial RNA purification kit, *Quick-RNA^TM^* MiniPrep (Zymo Research, Irvine, CA, USA). The manufacturer’s protocol was followed, and the purified viral RNA was eluted in 60 µL of RNase-free water.

#### 2.3.2. Reverse-Transcription

In the reverse-transcription step, to 10 µL out of the 60 µL of the extracted RNA were added 1 µL of 100 µM Random Hexamer primer, 1 µL dNTP mix (10 mM each of the four deoxynucleoside triphosphate stocks), 2.5 µL DEPC-treated water, 4 µL 5 × RT buffer, 0.5 µL Ribolock RNase inhibitor and 1 µL of 200-U/µl RevertAid^TM^ Premium reverse transcriptase (Fermentas, Burlington, ON, Canada) in the indicated order into a 0.5 mL PCR tube on ice. The reaction mixture (25 µL) was briefly vortexed to ensure total mixing and then centrifuged. The tubes were then incubated at 25 °C for 10 min followed by 30 min at 60 °C. The reaction was terminated by heating at 85 °C for 5 min. The resulting 20 µL of cDNA was kept at −20 °C until use for qPCR. For RoV, prior to reverse-transcription, the RNA was subjected to denaturation at 95 °C for 5min followed by flash chilling in ice for 2 min, to separate the double-stranded rotaviral RNA [59].

#### 2.3.3. Quantitative PCR (qPCR)

Briefly, 5 µL out of 20 µL of the cDNA was mixed with 20 µL of a reaction buffer (containing 12.5 µL of 2 × TaqMan universal PCR master mix (Applied Biosystems), 400 nM sense primer, 400 nM antisense primer, and 250 nM TaqMan probe and PCR grade water to give a 25-µL total reaction mixture [13]. Subsequently, the mixture was added to a well of a 96-well micro-plate and loaded into the *StepOnePlus* PCR System. Fluorescence data were collected at the end of annealing step. The thermal cycling protocols used for the respective viruses are shown in Table 2.

**Table 2:** The thermal cycling protocols for qPCR, using cDNA of the respective RNA viruses.

Virus	Taq activation	45 cycles of:		
		Denaturation	Annealing	Extension
Hepatitis A virus	10 min at 95 °C	15 s at 95 °C	1 min at 60 °C	1 min at 70 °C
Rotaviruses	15 min at 95 °C	15 s at 95 °C	30 s at 55 °C	30 s at 72 °C
Enteroviruses	10 min at 95 °C	15 s at 94 °C	1 min at 58 °C	20 s at 72 °C

#### 2.3.4. The Standard Curve

The standard curve was obtained as described by Haramoto *et al.* [58]. Briefly, the viral RNA was extracted from each positive control strain using *Quick-RNA^TM^* MiniPrep (Zymo Research). RNA extracts was then reverse-transcribed into cDNA. The cDNA was then quantified (in µg/mL) using a Qubit fluorometer (Invitrogen, Carlsbad, CA, USA) and diluted to 10 µg/mL, equivalent to 10 ng/µL. This quantity was then diluted by serial ten-fold dilution. Viral cDNA generated from the samples and standards were each run in duplicate and simultaneously. The runs were followed by analysis using SDS software (Applied Biosystems) to obtain quantitative data used to calculate the target gene concentration. A sample with threshold cycle (C_T_) value of ≤35 was defined as positive. The concentrations of the viruses in the river water samples from the studied sites are equivalent to the target gene copies per liter (*i.e.*, each HAV, RoV and EnV particle consists of one copy of the 5′ NCR, NSP3 and 5′ UTR gene, respectively).

### 2.4. Quality Control

Negative controls (non-spiked autoclaved distilled water) and positive controls (virus suspensions) were incorporated with each set of test samples and subjected to RNA extraction and PCR assays. To avoid the number of false-positives resulting from carryover contamination of the samples or amplified cDNA, virus control extractions were carried out using a safety cabinet. RNA extractions from water samples, reverse transcription of the extracted RNA into cDNA and PCR amplifications were performed in separate laboratory rooms. Viral reference strains (Table 1) were obtained from the American Type Culture Collection (ATCC, Manasas, VA, USA). All qPCR runs included a negative control reaction (PCR-grade H_2_O without template). To prove the specificity of the primer sets for the detection of human enteroviral viral particles, bovine enterovirus Type 1 (ATCC VR-248) was included as a negative control in the qPCR assay for enteroviruses. No internal amplification control (IAC) was included in these assays. The detection limit for each virus was determined to be 10 genome copies/litre. For accurate and precise measurements, a broad range of standard quantities (7 logs) were used to construct the standard curves for HAV (Y = −3.479x + 39.861; R^2^ = 0.970), RoV (Y = −3.359x + 18.483; R^2^ = 0.999) and EnV (Y = −3.809x + 33.261; R^2^ = 0.998). DNAZap^TM^ solution (Ambion^®^, Foster City, CA, USA), which completely degrades all RNA and DNA, was used to always wash the pipettes to prevent carryover contaminations.

### 2.5. Statistical Analysis

One-way ANOVA and Tukey’s test were carried out to test differences among the mean concentrations, using the Statistical Package for the Social Sciences (IBM SPSS Statistics release 19; IBM, Armonk, NY, USA). 

## 3. Results and Discussion

**Figure 2:**
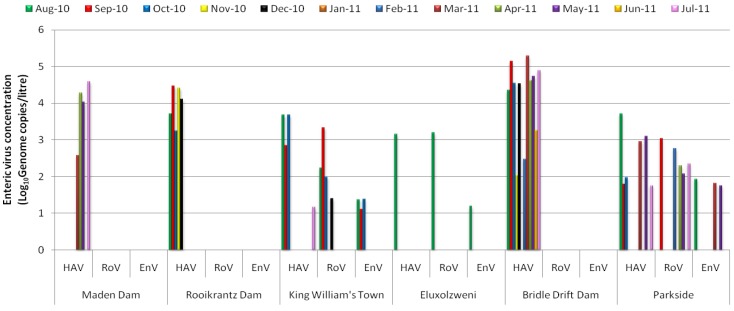
Spatial and monthly variations in concentrations of enteric RNA viruses (log_10_ genome copies/litre) in water samples collected from six sites in the Buffalo River.

**Figure 3:**
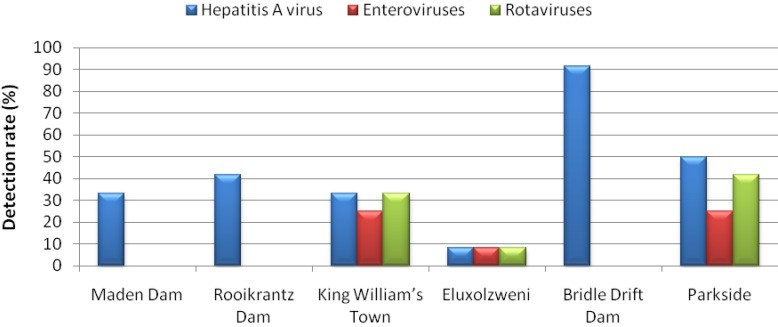
The detection rates for enteric viruses in the Buffalo River and at the six sampling sites along the Buffalo River.

Figure 2 shows the results for quantitative detection of HAV, RoV and EnV and reveals spatial and monthly variations in concentrations of these viruses (log_10_ genome copies/L) in water samples collected from six sites on the Buffalo River. Figure 3 shows the detection rate per site. Detection rates and viral concentrations varied with sites and virus, with significantly higher (*p* < 0.05) concentrations and very high detection rates being recorded for Bridle Drift Dam and HAV. While only HAV was detected at all the sites, it was only at King William’s Town, Eluxolzweni and Parkside that all three enteric RNA viruses studied were detected.

HAV was detected at all the sites and in all but one (January) of the 12 months in 43% (31/72) of the samples, with concentrations ranging from 1.5 × 10^1^–1.9 × 10^5^genome copies/L. This high detection rate is similar to a previous report from South Africa [22], in which HAV was detected in 35.3% of the river and 37.3% of the dam water samples tested. However, it is higher than that of a 2007 report in which Venter *et al*. [23] reported the detection of HAV in 17.5% of river and 14.9% of dam water samples they tested. The presence of HAV at all the sites may suggest that the virus is consistently present in Buffalo River. The Bridle Drift Dam samples yielded a detection rate (91.7%) with HAV concentrations ranging from 1.1 × 10^2^–1.9 × 10^5^genome copies/L. HAV was detected in 50% (6/12) of samples collected at Parkside, with concentrations ranging from 5.6 × 10^1^–5.2 × 10^3 ^genome copies/L, in 41.7% (5/12) at Rooikrantz Dam, with concentrations raging from 1.7 × 10^3^–2.9 × 10^4^ genome copies/L, in 33.3% (4/12) at Maden Dam, with concentrations ranging from 3.8 × 10^2^–3.9 × 10^4^ genome copies/L), in 33.3% (4/12) at King William’s Town, with concentrations ranging from 1.5 × 10^1^–4.8 × 10^3 ^genome copies/L and in 8.3% (1/12) at Eluxolzweni, with a concentration of 1.4 × 10^3 ^genome copies/L.

The highest detection rate of 91.7% for HAV observed at Bridle Drift Dam, with a mean concentration of 5.4 × 10^4^ genome copies/L, is not surprising. The dam is located in and downstream of densely populated areas and discharge points of sewage treatment works that are not very efficient [65,68]. HEntVs are excreted in high concentrations (10^5 ^to 10^13^/g faeces) in the faeces of infected persons, have great potential to pollute water sources [10], and the discharge of inadequately treated sewage effluents is the most common source of enteric viral pathogens in aquatic environments [8]. The high detection rates (33–42%) and mean concentrations that were observed for HAV at Maden Dam (1.72 × 10^4^ genome copies/L) and Rooikrantz Dam (1.49 × 10^4^ genome copies/L) are unexpected, considering the very low human population densities of the catchments of the two dams. However, HAV has been shown to cause acute hepatitis, not only in humans but also in some primates [36,69,70], and the bushy and mountainous catchments of the two dams were observed to host a high population of monkeys. The sequence alignment of target regions of the primers and probe used in this study proved them to be adequate for the quantification of all HAV genotypes [56]. Although the natural transmission of human HAV from experimentally infected animals to humans is well documented, the susceptibility of humans to true simian HAV strains is still unknown [71]. Should the HAV detected in this study (especially at both Maden and Rooikrantz dams) be simian genotypes, the waters from both dams therefore could represent no HAV-related risks for public health. There is need for genotypic characterization of the detected HAV which could differentiate between possible simian strains from the human strains.

HAV was detected more often in the winter and spring months. In general terms, the detection of hepatitis A virus in Buffalo River is similar to other studies in other provinces in South Africa [22,23] and elsewhere [54]. While the detection rate for HAV in this study was about 43% (31/72), in the study by Taylor *et al*. [22], HAV was detected in 18 (35.3%) of river and 19 (37.3%) of dam water samples, with a seasonal peak being evident in both the river and dam water in early spring (August and September). It was also during these two months that HAV was detected most often in this study. Although Taylor and colleagues detected HAV less often in the months of May, June and July, it was during the months of January and February that the least HAV detection was recorded in this study. Considering that we used real-time quantitative reverse transcriptase-polymerase chain reaction (RT-qPCR) which is more sensitive, compared with the combination of cell culture amplification and qualitative RT-PCR used by Taylor, the variations were not unexpected.

The RoV concentrations ranged from 2.5 × 10^1 ^genome copies/L to 2.1 × 10^3^genome copies/L and RoV were detected in 14% (10/72) of the samples (Figure 2). In a report by van Zyl *et al*. [47], on the molecular epidemiology of Group A rotaviruses in water sources and selected raw vegetables in Southern Africa, Group A RoV were detected in 11.8% of partially treated and 1.7% of finally treated drinking water samples; in 14% of irrigation water samples; and 1.7% of corresponding raw vegetable samples. However, higher detection rates have been reported elsewhere. Lodder *et al*. [72] reported the detection of RoV in 48% of the surface water samples in The Netherlands. The frequency of virus detection varied greatly between the locations. Fifty percent of RoV detections occurred at Parkside, 40% at King William’s Town, and 10% at Eluxolzweni. No RoV was evident at any of the three dams. This could be due to human population difference along the river course. The lower catchments are significantly more populated than the upper catchments. In a similar report from Kenya, Kiulia *et al*. [41] detected Group A rotavirus in 10 (100%) of samples collected from a river located in urban area and in three (25%) of rural river water samples. 

Several studies on the seasonality of rotavirus at various locations with different climatic conditions in South Africa identified two recurrent features of the disease. First, rotavirus infection occurred year-round in all locations studied; and secondly, in each region, rotavirus cases increased during the cooler and drier months [73]. In this study, RoVs were detected in each of the four seasons. Also, 80% of all RoV detections were made in the cooler months of August to October in 2010 and April to July in 2011. Only 20% of RoV detections occurred in summer months of December and February.

In this study, the detection rate for EnV was 9.7% (7/72), with concentrations that ranged from 1.3 × 10^1^genome copies/L to 8.6 × 10^1 ^genome copies/L. In a study that had been previously conducted in South Africa, Ehlers *et al*. [74], who used a combination of cell culture and nested-PCR, reported the presence of enteroviruses in 42.5% of sewage, 18.7% of treated drinking water, 28.5% of river water, 26.7% of dam/spring water and in 25.3% of borehole water samples. The detection rate of 9.7% observed for EnV in this study is unexpectedly low considering the high sensitivity of the RT-qPCR used in this study and reported high seroprevalence for enteroviruses in human populations [75].

While no EnV were detected during the hotter months (November 2010 to February 2011), 42.8% of all EnV detections were made in the winter month of August (Figure 2). This is consistent with previous studies that reported a significant correlation between low water temperatures and occurrence of enteroviruses in water [76]. EnV were detected in 9.7% (7/72) of the samples by real-time RT-PCR and in 1.4% (1/72) by semi-nested reverse transcriptase PCR, thus reaffirming the higher sensitivity of qRT-PCR in comparison with conventional RT-PCR [54]. The detection rate of 1.4%, by semi-nested PCR, recorded in this study is similar to the 1.2% reported elsewhere [52].

While about 57% (4/7) of the positive samples were from a combination of two freshwater sites (three samples from King William’s Town and one from Eluxolzweni), about 43% (3/7) of the EnV-positive samples were from the Buffalo estuary. This observation is consistent with reports that EnV are tolerant of a wide range of temperatures and salinities which facilitates their survival in environmental waters [57,77]. However, our findings apparently differ from the results presented by Schvoerer *et al*. [52], in which they studied 26 water samples in southwestern France, and detected EnV in freshwater, but not in seawater. A comparison of the detection rates of the three enteric RNA viruses assessed in this study (Figure 3) reveals that only HAV was detected at all the sites. Neither RoV nor EnV was detected at any of the dams. The mean concentration of HAV (2.5 × 10^4^ genome copies/L) detected in the Buffalo River was significantly higher (*p* ˂ 0.05) than that RoV (6.2 × 10^2^ genome copies/L) and EnV (4.0 × 10^1^ genome copies/L). This may reflect both the epidemiological status of infections caused by these viruses and there survival in the water environments.

## 4. Conclusions

Contamination of watercourses with enteric viruses represents a significant risk to public health. Although the RT-qPCR method used in this study does not directly test for viral infectivity, our results show that water samples from Buffalo River and the source water dams along its course were heavily contaminated with hepatitis A virus and to a lesser extent with rotaviruses and enteroviruses. The presence of HEntVs in water is therefore a potential health risk for the rural communities that use the water for domestic purposes and also the persons who swim in the river and dams or eat fresh-produce irrigated with water from these sources. The seriousness of the health effects from a viral infection will depend, however on the specific virus, as well as the characteristics of the individual affected (e.g., age, health status). The risk is heightened by the low infective doses of these viruses. Future research involving the genotypic characterization of the detected HAV could differentiate between possible simian strains from the human strains.
